# Genetic studies of Polish migraine patients: screening for causative mutations in four migraine-associated genes

**DOI:** 10.1186/s40246-015-0057-8

**Published:** 2016-01-08

**Authors:** Izabela Domitrz, Michalina Kosiorek, Cezary Żekanowski, Anna Kamińska

**Affiliations:** Department of Neurology, Warsaw Medical University, 61 Żwirki i Wigury Street, 02-091 Warsaw, Poland; Department of Neurodegenerative Disorders, Mossakowski Medical Research Centre PAS, 5 Pawinskiego Street, 02-106 Warsaw, Poland

**Keywords:** Familial hemiplegic migraine, Aura migraine, *SCN1A*, *KCNK18*, Missense mutations, Splice variants, SNP, Genetic background

## Abstract

**Background and aim:**

Migraine is the most common neurological disorder, affecting approximately 12 % of the adult population worldwide, caused by both environmental and genetic factors. Three causative genes have been identified in familial hemiplegic migraine (FHM) families: *CACNA1A*, *ATP1A2*, and *SCNA1A*. Recently, several mutations in *KCNK18* have also been found as causative factors in migraine development. The aim of our study was to identify the genetic background of migraine in the Polish population.

**Material and methods:**

Sixty patients with migraine without aura (MO) or with different types of migraine with aura (MA), including sporadic hemiplegic, familial hemiplegic, and probable familial hemiplegic, were screened for mutations in the four genes previously linked with different types of migraine (*ATP1A2*, *CACNA1A*, *SCN1A*, and *KCNK18*).

**Results:**

Two missense mutations were found. One novel mutation in *SCN1A*, encoding α subunit of sodium channel, causing amino acid change M1500V localized to a region encoding inactivation loop between transmembrane domains III and IV of the channel, was detected in a female FHM patient. The M1500V mutation was absent in a group of 62 controls, as well as in the ExAC database. The second, already known missense mutation S231P in *KCNK18* was found in a female MA patient. Additionally, a novel intronic polymorphism possibly affecting alternative splicing of *SCN1A*, at chr2:16685249, g.77659T>C, and c.4581+32A>G, located between exons 24 and 25, in a region encoding the inactivation loop of the sodium channel was found in a female MO patient. No mutations in *ATP1A2* or *CACNA1A* were found in the study group.

**Conclusions:**

The presence of *SCN1A* mutations and absence of mutations in *ATP1A2 or CACNA1A* suggest that the Polish patients represent FHM type 3. On the other hand, the presence of *KCNK18* mutation indicated another FHM subtype. It could be speculated that contrary to other European populations, the genetic basis of migraine in the Polish population involves mutations in genes not included in the study. Next-generation sequencing methods should be implemented to identify other migraine-associated variants.

**Electronic supplementary material:**

The online version of this article (doi:10.1186/s40246-015-0057-8) contains supplementary material, which is available to authorized users.

## Introduction

Migraine is the most common neurological disorder, affecting approximately 12 % of the adult population worldwide, caused by both environmental and genetic factors. Its heritability is estimated at ca. 40–70 %. Numerous linkage studies and candidate-gene studies have identified causative genes in migraine without aura (MO) and migraine with aura (MA). Three such genes have been identified in familial hemiplegic migraine (FHM). The first FHM type (FHM1; MIM141500) is related to *locus* 19p13 and caused by mutations in *CACNA1A*, encoding alpha 1A subunit of voltage-dependent calcium channel type P/Q (MIM601011) [1]. Mutations in *CACNA1A* contribute also to episodic ataxia (EA2; MIM108500) [2] and spinocerebellar ataxia type 6 (SCA6; MIM183086) [3]. The second FHM type (FHM2; MIM602481) is caused by mutations in *ATP1A2* encoding alpha 2 subunit of Na+/K+−ATPase (MIM182340) [4]. Finally, the third FHM *locus* (FHM3; MIM609634) is at 2q24, and the implicated gene *SCNA1A* (MIM182389) encodes α subunit of the neuronal voltage-gated Na+ channel [5]. Mutations in *SCN1A* were also recognized as a cause of epilepsy [6]. Recently, *KCNK18* at *locus* 10q25.3, encoding K+ channel subfamily K member 18, has been found to be connected with FHM classified as other than types 1, 2, and 3 (MIM613655) [7]. Other studies have provided evidence on the influence of the genetic variants of *SLC6A4* encoding a serotonin transporter on a migraine occurrence [8]. Moreover, recent genome-wide association studies (GWAS) have identified four novel genetic variants associated with migraine [9]: rs1835740 modulating glutamate homeostasis and specific for migraine with aura; rs11172113 implicating lipoprotein receptor LRP1, which may interact with neuronal glutamate receptors; rs10166942 in close proximity to *TRPM8*, encoding a cold and pain sensor; and rs2651899 in *PRDM16*, with an unclear role [9]. All these variants only confer a small to moderate risk for migraine, which concurs with migraine being a heterogeneous disorder [9]. A list of single nucleotide polymorphisms in 10 genes was reported in a large German case-control cohort study of migraine with aura [10], and recently, several genetic variants in *APOA1BP*, *TBC1D7*, *FUT9*, *STAT6*, *ATP5B*, *AJAP1*, *TSPAN2*, *FHL5*, *C7orf10*, and *MMP16* have been found in GWAS performed in Western Europe [11].

To identify the genetic background of migraine in Polish patients, we screened *ATP1A2*, *CACNA1A*, *SCN1A*, and *KCNK18* for mutations in a group of 60 patients with migraine without aura or with different types of aura migraine: typical, sporadic hemiplegic and familial hemiplegic or probable familial hemiplegic. All patients were Caucasians of Polish origin.

## Materials and methods

### Ethics statement

Written consent was obtained from probands, diagnosed family members, and probands’ relatives according to the Declaration of Helsinki [12]. The genetic study was approved by the Ethics Committee of the Medical University of Warsaw (Warsaw, Poland) in compliance with the national legislation and the Code of Ethical Principles for Medical Research Involving Human Subjects of the World Medical Association.

### Patients’ clinical description

Sixty patients (47 women and 13 men), mean age 48 ± 13 years, of the Headache Outpatient Clinic and Neurological Department, with different types of migraine diagnosed according to the International Headache Society (IHS) criteria, 3rd edition [13], were analyzed. The study group consisted of several subgroups: 4 families (Table 1—4a, b, c; 7a, b, c; 43a, b, c, d; 47a, b, c) with familial hemiplegic migraine (FHM3 families) and sporadic hemiplegic migraine (SHM1 family), 6 patients with probable familial hemiplegic migraine (F?HM—families of patients with hemiplegic migraine not seen), 7 patients with sporadic hemiplegic migraine (SHM), 11 patients with MA, and 23 patients with MO. Two members from FHM family no. 7 were also diagnosed with epilepsy with grand mal attacks (father and older son). One patient diagnosed SHM also had mental retardation. One patient with MO also had episodic ataxia (EA). Family no. 43 was examined for the presence of mutations in Leiden factor V. This analysis revealed the presence of the variant G1691A, c.1746G>A p.Arg534Gln, rs6025 in this family and in one family member (mother) was possibly associated with ischemic stroke. The study was performed at the Institute of Hematology and Blood Transfusion in Warsaw, according to standard protocols based on Sanger sequencing. Only the family 43 was examined for Leiden factor V genetic variants, and these tests were part of routine diagnostic practice at the Institute of Hematology and Blood Transfusion in Warsaw and were made available under standard medical care. The migraine patients were interviewed and examined in the Headache Outpatient Clinic or at the Department of Neurology of Warsaw Medical University. The diagnosis was performed by an experienced neurologist. Duration of the disease ranged between 1 and 40 years (mean duration 21 + 11 years), and the frequency of migraine attacks ranged between 1 attack/1 week to 1 attack/12 months. Patient profile is presented in Table 1. As a control group, we used our existing database of 62 samples of whole-exome sequencing, which were screened for the presence of identified variants.

**Table 1:** Clinical phenotypes and polymorphisms in migraine-related genes (*SCN1A*, *CACNA1A*, *ATP1A2*, *KCNK18)* in the study group

ID	Year of birth	Sex	Clinical phenotype	*SCN1A*, *CACNA1A*, *ATP1A2*, *KCNK18*
1	1980	M	MA	
2	1976	F	F?HM	c.4498A>G, p.M1500V, rs376885324 (ex. 24 SCN1A)
3	1978	F	MA	rs2298771 (ex. 16 SCN1A)
4a	1952	F	FHM	rs17846715 (ex. 16 ATP1A2)
4b	1978	F	FHM	
4c	1980	F	FHM	
5	1967	F	MO	
6	1967	F	MO	
7a	1954	M	FHM + EPI	
7b	1982	M	FHM + EPI	rs2298771 (ex. 16 SCN1A), rs16012 (ex. 13 CACNA1A)
7c	1984	M	MO	rs2298771 (ex. 16 SCN1A)
8	1980	M	SHM	
9	1958	F	F?HM	
10	1944	F	MO	Novel intronic polymorphism c.4581 + 32A>G, between ex. 24 and 25
11	1946	F	MO	
12	1954	F	MO	
13	1982	F	MA	
14	1966	F	MO	rs2298771 (ex. 16 SCN1A)
15	1962	M	MO	
16	1972	F	MA	c.691T>C, p.S231P, rs363315 (ex. 3 KCNK18), rs16016 (ex. 16 CACNA1A)
17	1962	M	MO	
18	1968	F	MO	
19	1943	F	MO	rs2298771 (ex. 16 SCN1A)
20	1963	F	F?HM	rs41288127, rs55884181 (ex. 22 ATP1A2)
21	1957	F	F?HM	
22	1978	F	MO	rs2298771 (ex. 16 SCN1A)
23	1968	F	MA	rs2298771 (ex. 16 SCN1A)
24	1934	F	MO	rs2298771 (ex. 16 SCN1A)
25	1960	F	MO	
26	1953	F	MA	rs16012 (ex. 13 CACNA1A)
27	1973	F	SHM	rs41276894 (ex. 4 CACNA1A)
28	1977	F	F?HM	
29	1988	M	SHM + MR	
30	1969	F	F?HM	rs61734524 (ex. 9 ATP1A2)
31	1973	F	SHM	rs17846715 (ex. 16 ATP1A2)
32	1976	M	MO	rs2298771 (ex. 16 SCN1A)
33	1987	F	SHM	rs17846715 (ex. 16 ATP1A2), rs41288127 (ex. 22 ATP1A2)
34	1970	F	MO	rs2298771 (ex. 16 SCN1A)
35	1972	F	MO	
36	1968	F	MO	
37	1966	F	MA	rs2298771 (ex. 16 SCN1A)
38	1969	F	MA	
39a	1988	F	MO	
39b	1960	F	MO	
40	1970	F	MO	
41	1974	F	MO	
42	1958	F	MO	
43a	1989	M	MO (mutation in Leiden factor V)	rs61734524 (ex.9 ATP1A2), rs16016 (ex. 16 CACNA1A)
43b	1994	F	FHM (mutation in factor V Leiden)	rs61734524 (ex.9 ATP1A2)
43c	1970	F	FHM (mutation in factor V Leiden)	rs61734524 (ex.9 ATP1A2), rs16016 (ex. 16 CACNA1A)
43d	1950	M	Ischemic stroke (mutation in factor V Leiden), mother with FHM + MA	
44	1958	F	MO + EA	
45	1960	F	MA	
46a	1943	F	SHM mother	rs61734524 (ex. 9 ATP1A2)
46b	1984	M	SHM	Not tested
46c	1943	M	SHM father	
47	1962	F	MA	rs17846715 (ex. 16 ATP1A2)
48	1967	M	SHM	
49	1963	F	MA	
50	1972	F	SHM	rs61734524 (ex. 9 ATP1A2), rs17846715 (ex. 16 ATP1A2)
51	1959	F	MA	rs2298771 (ex. 16 SCN1A)

Description of clinical phenotype: *EA*, episodic ataxia, *Epi* epileptic seizures, *F?HM* probable familial hemiplegic migraine, *FHM* familial hemiplegic migraine, *MA* migraine with typical aura, *MO* migraine without aura, *MR* mental retardation, *SHM* sporadic hemiplegic migraine

### Sanger sequencing

Genomic DNA was extracted from peripheral blood leukocytes using standard salting-out method. Exons 4, 5, 13, 16, 17, 24, 26, 29, 32, and 36 of *CACNA1A*, exons 6, 14, 15, 16, 18, 20, 23, 24, and 26 of *SCNA1A*, exons 9, 16, 17, 18, 19, and 22 of *ATP1A2*, and exons 1, 2, and 3 of *KCN1K18* (all with flanking intronic sequences) were sequenced using automated, fluorescent sequencing method. Genes and the exons were chosen on the basis of the frequencies of known migraine-causing mutations (Leiden Familial Hemiplegic Migraine Variation Database). In brief, the amplicons were amplified with Master Mix Kit and Hot Start Master Mix Kit (Qiagen) using genomic DNA from each patient. Primers were designed using Primer Premier software (Additional file 1). The annealing temperature of 58 °C was applied for all primers. Exon 26 of *SCN1A* and exon 3 of *KCNK18* were amplified in overlapping fragments. Resulting amplicons were purified with Exonuclease I/FastAP (Fermentas) and sequenced using ABI PRISM 3130 Genetic Analyzer (Applied Biosystems) with the BigDye Terminator v1.1 Cycle Sequencing Kit (Applied Biosystems).

## Results

### *SCN1A*

**Fig. 1:**
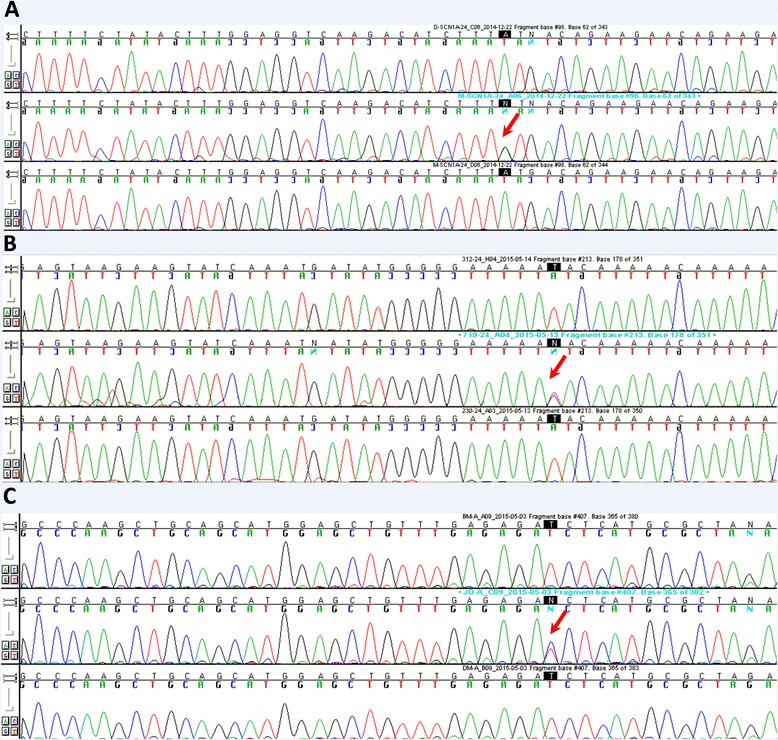
Mutations in migraine-causing genes. **a** Single nucleotide substitution at c.4498 A>G, resulting in novel amino acid change M1500V, was found in *SCN1A* in a patient with FHM (rs121918632, NG_011906.1, Gene ID: 6323). **b** A novel intronic polymorphism at chr2:16685249, g.77659T>C, c.4581 + 32A>G, located between exons 24 and 25, predicted to affect alternative splicing of *SCN1A*, in a gene region encoding inactivation loop of sodium channel was found in a patient with MO. **c** Mutation at c.691T>C, resulting in amino acid change S231P, in *KCNK18* (rs363315, NG_028085.1, Gene ID 338567) was found in a patient with MA

**Fig. 2:**
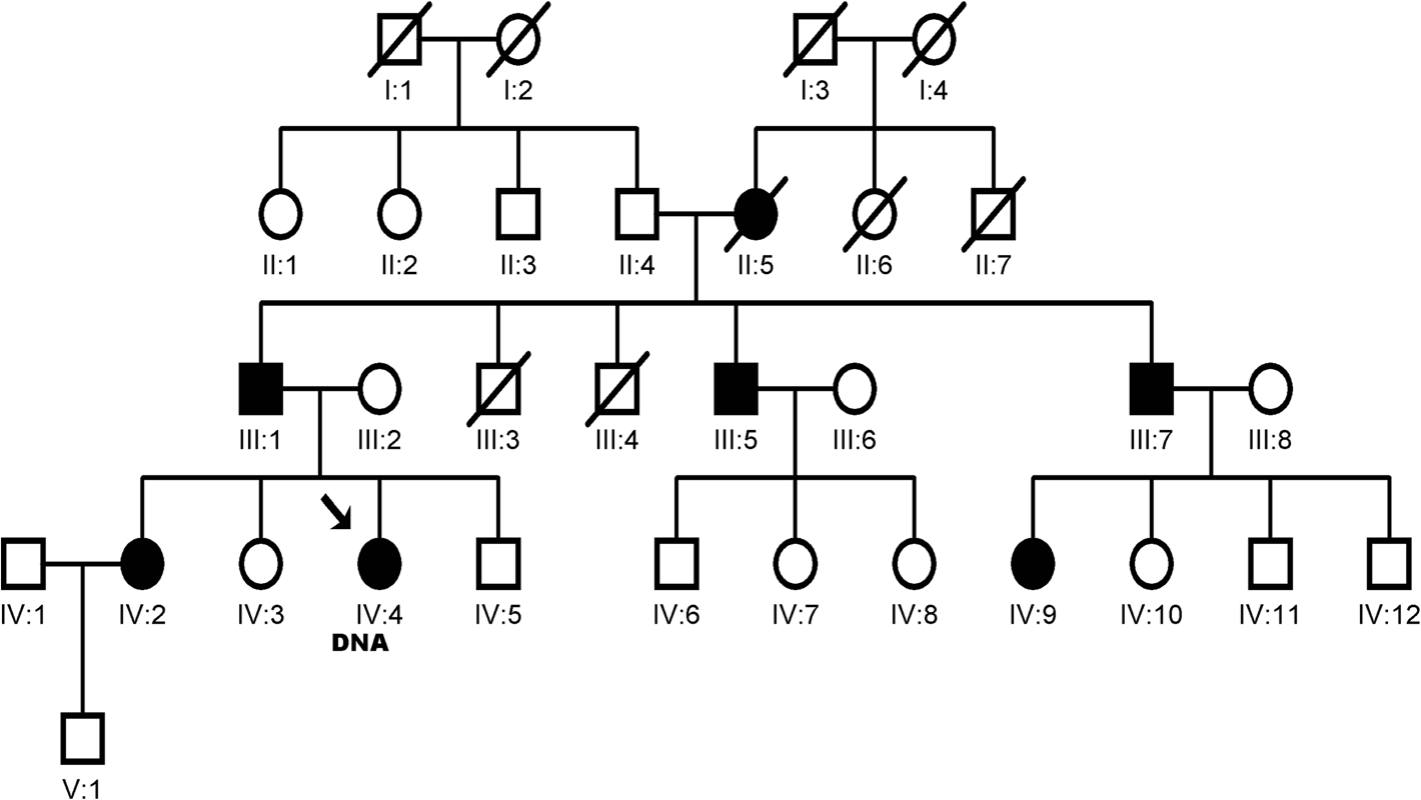
Pedigree of FHM patient with M1500V mutation in exon 24 of *SCN1A*

Among the 60 Polish patients studied, we found a single nucleotide polymorphism c.4498A>G, rs376885324, in exon 24 of *SCN1A* leading to amino acid substitution M1500V (Fig. 1a). This variant was found in a female FHM patient and had not been linked to an FHM clinical phenotype before (see Table 2). The family history of the patient suggested autosomal dominant inheritance pattern, and according to clinical interview, seven members of the proband’s family were also affected by migraine, including a sister, the father, two father’s brothers, a cousin, and the grandmother (Fig. 2). Unfortunately, the material for genetic testing from other family members was unavailable, but the patient’s sister and father were examined clinically and diagnosed with FHM in our clinic.

**Table 2:** Clinical phenotype of FHM patient with mutation M1500V in exon 24 of *SCN1A*

Hemiplegic migraine (HM) onset (years)	22
Triggering factors of HM	Emotional stress
Hemiplegia during aura phase	+
Sensory disturbances during aura phase	+
Visual disturbances during aura phase	+
Aphasia during aura phase	+
Aura duration (min)	60
Headache duration (h)	4–24
Side of headache and hemiplegia	Bilateral—variable sides
Character	Pulsating
Nausea/vomiting/photophobia/phonophobia during migraine attack	+/−/−/−
Other types of headache	MO, MA

Bioinformatic assessment using PolyPhen-2 HumDiv [14] showed that the M1500V variant is probably damaging with a score of 0.982 (sensitivity, 0.75; specificity, 0.96). According to the Protein Variation Effect Analyzer (PROVEAN) and Sorting Intolerant From Tolerant (SIFT) tools [15, 16], the variant is damaging (score −3.68) and deleterious (score 0.001). The MutationTaster prediction tool [17] also describes the variant as disease-causing. M1500V was not detected in our 62 controls or in the ExAC database, which contains data for the genomic region of interest from 60,000 DNA samples, including more than 30,000 samples from European populations. However, the mutation is present in the Ensembl database with a frequency of 1:8593.

**Fig. 3:**
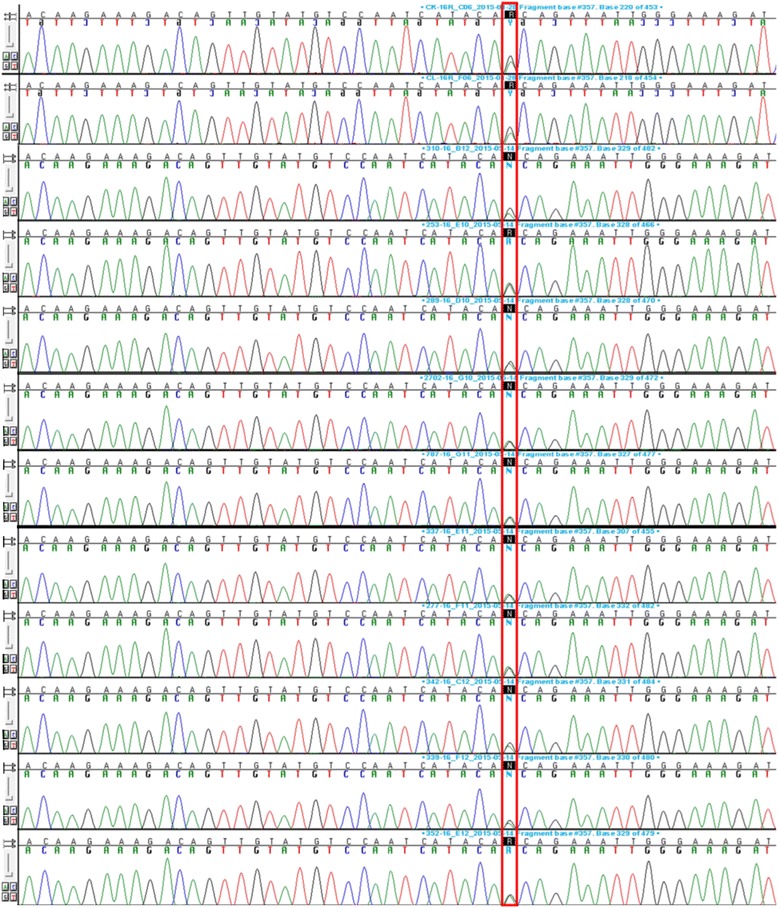
A1067T mutation in *SCN1A*. Exon 16 with flanking intronic regions of *SCN1A* gene was analyzed as described in the “Materials and methods” section. The missense variant at chr2:166892788, g.37362G>A, c.3199G>A, rs2298771 resulting in amino acid change A1067T found in 12 patients with different phenotypes (20 % of the study group) and present at the high frequency of 24 % in dbSNP database is indicated by frame

In addition to the M1500V mutation, we identified several synonymous single nucleotide polymorphisms in *SCN1A* in the study group and one missense variant at chr2:166892788, g.37362G>A, c.3199G>A, rs2298771, which the frequency of occurrence, according to data from the dbSNP database, is 24 %, and in our study group was 20 % (in 12 patients with different phenotypes), resulting in amino acid change A1067T (Table 1, Fig. 3). According to PROVEAN and SIFT, this amino acid substitution is neutral with a score of −0.068, and according to PolyPhen-2 mutation, is benign with a score of 0.000. The MutationTaster scored the variant as a neutral polymorphism that might change protein feature. This variant has been assigned to myoclonic astatic epilepsy phenotype [18], which is in accordance with our study showing its presence in one family (no. 7; three patients, 5 % of the study group) with epilepsy and FHM.

In a female patient with migraine without aura and with onset at puberty, we found a novel intronic polymorphism possibly affecting alternative splicing of *SCN1A*, at 2:166852491, g.77659T>C, c.4581+32A>G, located between exons 24 and 25, in a region encoding inactivation loop of the channel (Fig. 1b). The patient had migraine with a typical course: high-intensity attacks lasting for a few hours with frequency 1 per month. The patient’s familial history is surprising. Her two sisters were diagnosed with a probable CADASIL (cerebral autosomal dominant arteriopathy with subcortical infarcts and leukoencephalopathy), but with no mutation in *NOTCH3*. One of the sisters suffered from dementia and was diagnosed with disseminated and diffuse changes in brain MRI interpreted as the beginning of demyelination (she died at the age of 60 years, and no medical records were available). The patient’s mother died at the age of 69, likely from a stroke. The patient’s father and brother and the patient’s siblings’ children are healthy. The variant could not be evaluated by the PROVEAN, SIFT, or Poly-Phen2 tools due to a lack of data. This variant is absent in the ExAC database, but insertion A/AT has been found at this nucleotide position (http://exac.broadinstitute.org/region/2-166852491-166852491), with a frequency of ca. 0.000008. The substitution reported here could be a novel rare intronic variant. According to the MutationTaster, the variant could alter various protein features, including domains and motifs, downstream of the altered splice site, as presented in Table 3.

**Table 3:** Predicted loss of protein features downstream of the splice site altered by c.4581+32A>G variant in *SCN1A*

Start (aa)	End (aa)	Feature	Details
1523	1821	Repeat	IV
1537	1537	Conflict	F ->L (in [7]; CAA46439/M91803).
1537	1560	Transmem	Helical; name = S1 of repeat IV; (by similarity).
1572	1595	Transmem	Helical; name = S2 of repeat IV; (by similarity).
1602	1625	Transmem	Helical; name = S3 of repeat IV; (by similarity).
1636	1657	Transmem	Helical; voltage sensor; name = S4 of repeat IV; (by similarity).
1673	1695	Transmem	Helical; name = S5 of repeat IV; (by similarity).
1762	1786	Transmem	Helical; name = S6 of repeat IV; (by similarity).
1788	1788	Carbohyd	N-linked (GlcNAc…) (potential).
1915	1944	Domain	IQ

### *CACNA1A* and *ATP1A2*

We found several synonymous single nucleotide polymorphisms in the *CACNA1A* and *ATP1A2* genes, as summarized in a Table 1.

### *KCNK18*

We identified a known mutation at c.691T>C, rs363315, in exon 3 of *KCNK18* gene leading to amino acid substitution S231P in a female patient diagnosed with migraine with aura (Fig. 1c).

Additionally, in our study group, one family (no. 43; four patients, 6.6 % of the study group) had mutation in Leiden factor V, associated with blood clots. In this family, we did not find any mutations in *SCNA1A*, *CACN1A*, *KCNK18*, and *ATP1A2* reported before as migraine-causing.

## Discussion

Migraine is the most common neurological disorder with a well-defined genetic background, but until now, no migraine-causing mutations have been studied in Polish patients [19, 20]. For genetic screenings in this study, we have selected exons 4, 5, 13, 16, 17, 24, 26, 29, 32, and 36 of CACNA1A; exons 6, 14, 15, 16, 17, 23, 24, and 26 of SCNA1A; exons 9, 16, 17, 18, 19, and 22 of ATP1A2; and exons 1, 2, and 3 of KCN1K18 (all with flanking intronic sequences). We have selected these genetic regions according to the frequencies of found disease-causing genetic variants stated in Leiden Familial Hemiplegic Migraine Variation Database and to the highest probability of genetic variant occurrence.

Overall, our study sheds the first light on the genetic background of migraine in the group of Polish patients, and we believe that this study will help us to stratify our pool of patients in terms of their genetic status of the most common variants of the four genes associated with migraine for future large-scale genetic studies.

We have identified a novel FHM-linked M1500V mutation in *SCN1A*, putatively affecting the inactivation loop of the sodium channel protein and confirming previous reports found the S231P mutation in *KCNK18* in a patient with migraine with aura. Surprisingly, no disease-causing variants in *ATP1A2* and *CACNA1A* were found in the study group.

**Fig. 4:**
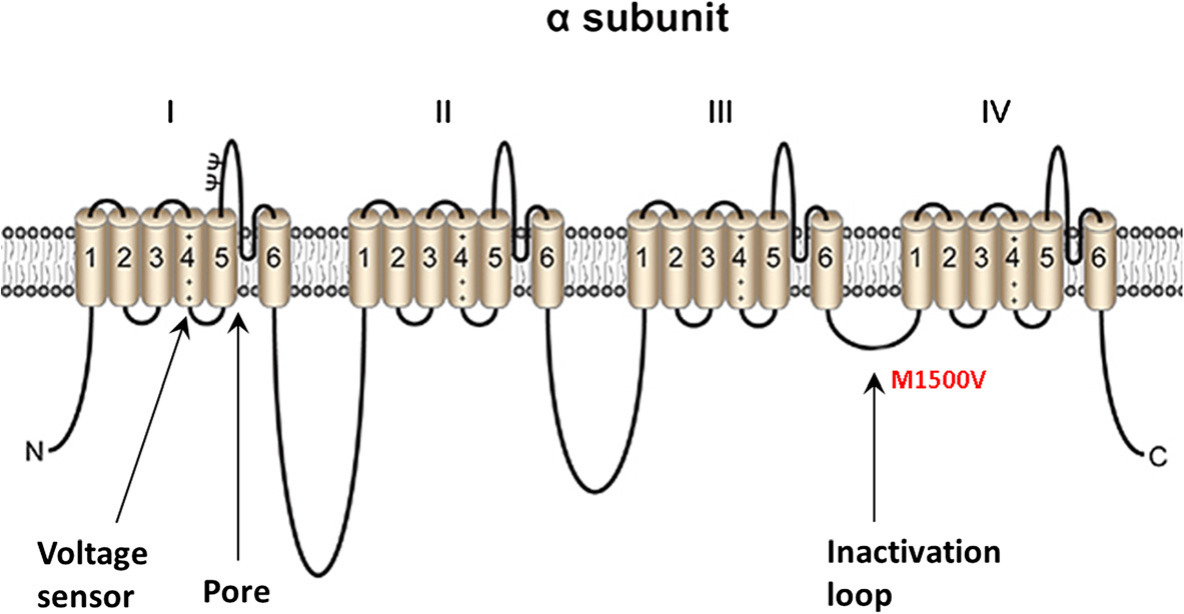
Transmembrane organization of sodium channel α subunit. The alpha subunit of the channel comprises four repeat domains (I–IV), each containing six membrane-spanning segments (1–6). The highly conserved segment 4 acts as the voltage sensor of the channel close to the pore for ion entry. After Na+ has entered the cell and the membrane’s potential reached its maximum, the channel inactivate by closing the inactivation loop. Mutation M1500V could abolish channel inactivation and lead to its constitutive activity and firing of the synapses; however, further functional studies are needed to verify this assumption. Adapted from Brackenbury and Isom [29]

The novel mutation M1500V substitutes evolutionarily highly conserved methionine residue in sodium channel protein type 1 subunit alpha (P35498). The functionally important amphipathic methionine involved in playing binding/recognition of hydrophobic ligand methionine is substituted with hydrophobic valine with a largely non-reactive aliphatic side chains and packed in the protein interior. There is no direct data indicating that M1500V is causally linked to the disease, but it localizes next to the known F1499L mutation (dbSNP:rs121918632), involved in episodes of elicited repetitive daily blindness (ERDB) co-segregating with familial hemiplegic migraines [21] and with I1498M [22]. The Met > Val substitution is in the IFM motif responsible for fast channel inactivation, located in the inactivation intracellular gate between domains III and IV, each composed of six transmembrane segments called S1–S6 (Fig. 4). Since normal fast inactivation of the channel results from occlusion of the intracellular part of the channel by a short loop between domains III and IV, mutation in this region could disturb the inactivation. For example, in oocyte expression studies, R1648H accelerated recovery from inactivation, W1204R shifted the voltage-dependence of activation and inactivation in the negative direction, R859C shifted the voltage-dependence of activation in the positive direction, and T875M enhanced slow inactivation [23]. The alterations due to R1648H and W1204R are predicted to increase sodium channel activity and neuronal excitability, whereas those due to R859C and T875M should decrease the channel activity and neuronal excitability [23]. Further, in vitro studies are necessary to assess the impact of the M1500V mutation on sodium channel inactivation.

The second mutation we found is S231P in *KCNK18*. This mutation has already been well characterized and shown to be migraine-related but not to influence the activity of the TRESK K2P potassium channel encoded by *KSNK18* [24]. Other studies on this variant also showed relationship to migraine with typical aura [25]. Accordingly, we found the *KCNK18* mutation in a patient with migraine without aura.

The novel intronic polymorphism identified in a family with a CADASIL-like phenotype and migraine could affect the splicing of *SCN1A* transcript. According to the MutationTaster, this variant may influence splicing (e.g., increase or gain of splice donor site at position g.77658) and affect protein features (for details, see Table 3). Voltage-dependent inactivation of Na+ channels is a consequence of voltage-dependent activation, and an inactivated Na+ channel enters a non-conducting and non-transmitting signals state, as the inactivation gate, the cytoplasmic loop linking domains III and IV of the α subunit, obstructs the open pore [26]. Due to its the location in the exon 24, encoding the inactivation loop, the variant seems likely to produce a constitutively inactive form of sodium channel alpha subunit leading to the permanent shut down of synaptic transmission. Detailed functional studies are needed to elucidate the link between generation of various alternative splicing forms and biological pathomechanisms and clinical phenotype of migraine.

Additionally, based on our studies, it could be speculated that the factor Leiden V mutation found in one family carrying no causative mutations in *SCNA1A*, *CACN1A*, *KCNK18*, or *ATP1A2* is a sole cause of migraine with aura and vision disability and strokes in that family, in line with several reports showing a linkage between migraine and Leiden factor variants [27, 28].

### Future perspectives

We suggest that mutations located in other parts of the analyzed genes or in other genes, not yet shown to be connected with migraine, cause various clinical forms of the disease in the Polish population. We postulate that next-generation sequencing (NGS) methods, e.g., whole-exome sequencing or whole-genome sequencing, should be used to identify comprehensively migraine-causative mutations. This is reinforced by the fact that recent large-scale studies from European populations have provided an extensive list of susceptibility genes linked to migraine. The list of candidate genes linked with FHM occurrence includes: *APOA1BP*, *TBC1D7*, *FUT9*, *STAT6*, *ATP5B*, *AJAP1*, *TSPAN2*, *FHL5*, *C7orf10*, and *MMP16* [11].

## Additional file

Additional file 1:
**Primers for selected exons of CACNA1A, SCN1A, ATP1A2 and KCNK18 gene designed using Primer Premier software.** (DOCX 23.2 kb)
