# Supplementary material for: Genetic studies of Polish migraine patients: screening for causative mutations in four migraine-associated genes
Source: Hum Genomics. 2016 Jan 8;10:3. doi: 10.1186/s40246-015-0057-8 (PMC4706665; doi:10.1186/s40246-015-0057-8)
Supplement: Additional file 1: — Primers for selected exons of CACNA1A, SCN1A, ATP1A2 and KCNK18 gene designed using Primer Premier software. (DOCX 23.2 kb) [file 40246_2015_57_MOESM1_ESM.docx]

**Aditional file 1.** Primers for selected exons of *CACNA1A, SCN1A, ATP1A2* and *KCNK18 gene* designed using Primer Premier software.

| gene | exon | 5'- 3' sequence | Strand | Tm (°C) | Product lenght (bp) |
| --- | --- | --- | --- | --- | --- |
| *CACNA1A* | 4 | AAACCCACCCTCTGTTCTCC | F | 58.8 | 208 |
|  |  | TGCTCTGCATAGGGGAAACT | R | 56.8 |  |
|  | 5 | TTATGGGGCCTCTCTCTGTG | F | 58.8 | 241 |
|  |  | CCAAGAGCTTGTCCCTGAAG | R | 58.8 |  |
|  | 13 | CACTGTCCCTGGACATGAGA | F | 58.8 | 235 |
|  |  | CCACCCCCTGTACAAATGTC | R | 58.8 |  |
|  | 16 | GGGTTGGCTGTGTGTTTCTT | F | 56.8 | 235 |
|  |  | CTCAGTACCCTCCCTTGAGC | R | 60.9 |  |
|  | 17 | CACCAGTGGTTGCTTTTCCT | F | 56.8 | 221 |
|  |  | TTCTAACCCTGTTCCCATGC | R | 56.8 |  |
|  | 25 | CTTCTAGGCCTGGGAGGGTA | F | 60.9 | 235 |
|  |  | ACCCCACCATCTCCCAAT | R | 55.3 |  |
|  | 26 | AGCCCCTCATGCTCTCTGT | F | 58.2 | 227 |
|  |  | AATGGGAATGTGCTGGAAAG | R | 54.7 |  |
|  | 29 | CTCTCACCACTGGCCTCTCT | F | 60.9 | 241 |
|  |  | GAACGAGGTGGGGGTTAAGT | R | 58.8 |  |
|  | 32 | CACCCTCTCTCTCCCAACAT | F | 58.8 | 369 |
|  |  | GCATGAGGGTCACCTGTCTT | R | 58.8 |  |
|  | 36 | TCATTCATTCCCTCGGTCTC | F | 56.8 | 228 |
|  |  | CAGTTCCAGGGAGAGGTGAG | R | 60.9 |  |
| *SCN1A* | 6 | TGTGAAGCTTTTATTATTTCACACG | F | 56.1 | 396 |
|  |  | TCCTTTGTGTTACAAACAATCCA | R | 54.9 |  |
|  | 14 | TGTGGGAAAATAGCATAAGCA | F | 57.3 | 338 |
|  |  | TCAGGGTTCTTGCATAGCAA | R | 56.9 |  |
|  | 15 | ACGGTTAGGGCAGATCAGAT | F | 57.8 | 660 |
|  |  | ACATTGTGCACATGTACCCT | R | 57.7 |  |
|  | 16 | TGTAATCTGCTCTTCCCTACATT | F | 60.1 | 569 |
|  |  | AGATTCTATAGGTGTGGGAAGGT | R | 59.6 |  |
|  | 18 | TGGCAGGCAACTTATTACCAA | F | 59.3 | 238 |
|  |  | CAGCTAAACAAGCTGCACTCC | R | 58.9 |  |
|  | 20a | CCATCTGGGCTCATAAACTTGT | F | 57.6 | 480 |
|  |  | AATTTTCAGCATCATGGGCG | R | 57.3 |  |
|  | 20b | TTAGGTGGTTGTGAATGCCC | F | 56.3 | 438 |
|  |  | AGTACAAGTTTATGAGCCCAGAT | R | 56.7 |  |
|  | 23 | AGCACCAGTGACATTTCCAG | F | 58.7 | 238 |
|  |  | TCTCAGTGGGAGAGAAAATATTAGA | R | 58.7 |  |
|  | 24 | GGACACAGTTTTAACCAGTTTGA | F | 56.5 | 370 |
|  |  | TCTTGAAGTCAAGGGTGTATCTTC | R | 56.1 |  |
|  | 26a | CGCATGATTTCTTCACTGGTT | F | 58.2 | 371 |
|  |  | CCAGCCAGCAGAGGTTGTA | R | 58.3 |  |
|  | 26b | GCAACAGCATGATCTGCCTA | F | 53.9 | 371 |
|  |  | CGCAGCTGCAAACTGAGATA | R | 54.6 |  |
|  | 26c | TCAGTTCATGGAATTTGAAAAA | F | 54.3 | 297 |
|  |  | GCAGATACTTCCTCTTGTTTTCG | R | 55.1 |  |
|  | 26d | TTCATGGCTTCCAATCCTTC | F | 59.3 | 400 |
|  |  | CAGGCTGTAAACAATTTGTCACC | R | 59.2 |  |
| *ATP1A2* | 9 | GAGCCACGGTCTAGGGTAAG | F | 55.5 | 356 |
|  |  | ATCTCAACCACTCACCCCAC | R | 59.4 |  |
|  | 16 | AAGGGGTTTCGTCCTCAAGT | F | 56.4 | 245 |
|  |  | ATCCTGCAAACCATCCCAAC | R | 56.8 |  |
|  | 17 | TGATGCCCTCAGAATCTCCC | F | 57.8 | 420 |
|  |  | CATCACGATTGCCTTGCCTG | R | 57.2 |  |
|  | 18 | TACGTCCCTTCAAATGCCCT | F | 56.7 | 247 |
|  |  | CTTGGGGCTCATTCCTCTGA | R | 55.6 |  |
|  | 19 | TTCCTGCTCTGACCCTGC | F | 55.3 | 244 |
|  |  | TTCAGCTTCCCGTACCTTCA | R | 55.5 |  |
|  | 22 | CAACCTCTGATGCTGCTGAC | F | 58.2 | 360 |
|  |  | AGGAACAGAAGCAGGAACCA | R | 57.8 |  |
| *KCNK18* | 1 | TTCCACCAGCTCCTCTTGC | F | 56.8 | 302 |
|  |  | ACGGAGAAAAGGCCCACC | R | 56.8 |  |
|  | 2 | GGGCTTGTCTTTACCAGCAG | F | 51.3 | 208 |
|  |  | ACTGTGGCTTTGCACTTACC | R | 50.5 |  |
|  | 3a | AAAAGGGAAGGGGCCAGATG | F | 58.5 | 483 |
|  |  | TGAGAGTCTTCCCAACACCA | R | 57.9 |  |
|  | 3b | CACAAGCCATGGAGAGGAGT | F | 54.7 | 499 |
|  |  | GCTGCATGACCCTGAAAGAC | R | 54.5 |  |
